# Motivations and market solutions for flexible housing in Finland

**DOI:** 10.1007/s10901-023-10013-5

**Published:** 2023-02-20

**Authors:** Rita Lavikka, Satu Paiho

**Affiliations:** VTT Technical Research Centre of Finland Ltd, P.O. Box 1000, 02044 Espoo, Finland

**Keywords:** Housing, Flexibility, Modularity, Multifunctionality, Sustainability

## Abstract

Flexibility is essential for sustainable housing and has been one of the design elements in Finnish architecture. However, between 1990 and 2010, flexible solutions in residential buildings were rare and included in only some advanced builders’ projects. Research on flexible housing exists but is scarce on knowledge of the 2020s drivers and market solutions for flexible housing. Therefore, we searched for trends, patents and market solutions for flexible housing in Finland. We also interviewed representatives of construction companies, designers, housing providers, financers and regulatory authorities to understand their views on flexibility; its meaning, benefits, challenges, market demand and technical solutions providing flexibility. We discovered several trends leading to flexibility in housing, e.g., urbanization and remote working, although no evidence of flexibility as a separate housing trend was found. We sought market examples for each trend to prove the markets’ potential interest in them. We found that the market need for flexible apartment buildings is currently low, even though the benefits of flexibility exist. However, market demand may increase if awareness of flexible options increases. No insurmountable technical challenges for housing flexibility exist, although the building services flexibility is complex. Flexible housing design, construction and solutions tend to cost more than a regular home. Flexibility in apartment buildings means multifunctionality inside a dwelling, using movable partitions and furniture or the ability to unite or separate two dwellings structurally. Modular construction is used to build these apartment buildings, supporting sustainability. Transferable and multifunctional wooden houses represent flexibility in small houses.

## Introduction

**Fig. 1:**
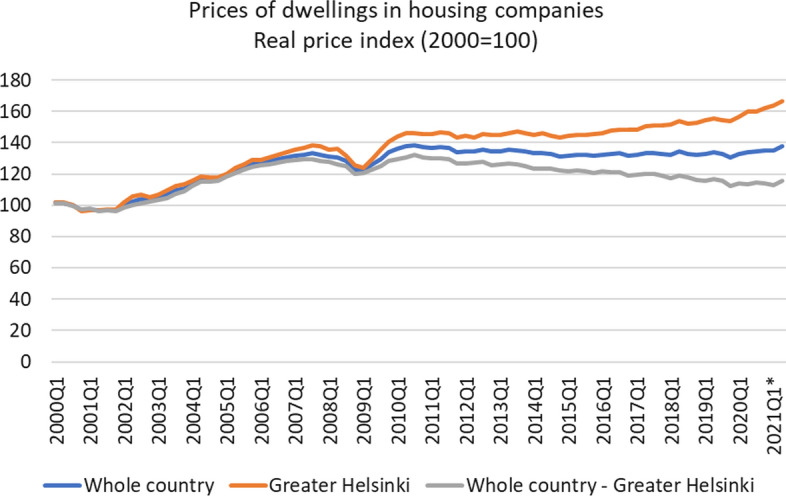
Quarterly price development of dwellings in housing companies in Finland. Data retrieved from (Statistics Finland, 2021a)

An increasing number of people are experiencing difficulties obtaining housing in Europe (Glumac, 2021; UNECO. Housing Europe, 2021). In Finland, the price development of housing has been growing, especially in the Helsinki capital area (Fig. 1). Furthermore, about 1.2 million people (almost a quarter of the whole population) live alone, and the number is increasing (Statistics Finland, 2021b), which partly emphasizes the need for smaller housing (Ruuskanen et al., 2019). In addition, the people in Finland are aging. It is estimated that by 2040, the number of people over the age of 65 will increase by 18% from the current just under 1.3 million (Statistics Finland, 2021b). At the same time, the number of people under 15 will fall by 13% from around 850,000 and the number of people aged 15–64 by 2% from the current 3.4 million (Statistics Finland, 2021b).

The statistical data emphasizes changing housing needs, partly due to rising prices and demographic changes. Also, the traditional concept of buying a house and living in it as long as possible is changing as some young people do not want to follow the owning tradition and be tied to one place, but lease or share a house, following living as a service concept (NREP, 2021). Covid-19 increased remote working, but many houses do not have enough space for a separate working room (PwC & the Urban Land Institute, 2022). Therefore, houses should be designed to be flexible to timely meet the multiple user needs and changes in the environment (Cellucci & Di Sivo, 2015; Estaji, 2017). Housing should be more flexible, especially during use. Entire homes or housing units should be flexibly transferable from one location to another. Sometimes such housing is referred being relocatable (e.g., Kyrö et al., 2019) or portable (e.g., Glumac, 2021). In the US, the terms manufactured or mobile homes or trailers are also used (Durst & Sullivan, 2019).

Additionally, all housing should support sustainability. Buildings and construction consume half of all extracted materials and produce approximately 5–12% of the total national greenhouse gasses (GHG) emissions (European Commission, 2021a; Eurostat—Statistical Office of the European Communities, 2021). Tarpio (2015) notes that flexibility during building use (functional adaptability) and its end of life (easily recyclable building parts) supports sustainable development through a higher use rate and long use phase of the building. For example, flexibility reduces construction waste and the environmental impact of new construction, such as the consumption of natural raw materials. The flexible dwelling’s space structure also enhances socially sustainable development when the residents can participate in the dwelling’s re-design to adapt to changing life situations (family size changes). The property owner benefits from functional adaptability by reducing life cycle costs (Tarpio, 2015).

Research shows that housing flexibility can benefit building owners and society by improving economic profitability by easier renting and saving building materials (Häkkinen & Ala-Kotila, 2019). Housing flexibility, however, usually costs more than the traditional solution during the construction phase. Thus, the realization of the benefits requires the application of flexible solutions during the life cycle of the building. Furthermore, it is argued that the knowledge of the flexibility solutions is not always available during the use and maintenance phases of the building, which prevents housing flexibility (Hakaste, 2014).

Flexibility in use has been one of the core design elements in Finnish residential architecture. For example, log buildings could be adapted for multiple users, activities and changes in external circumstances since each room was equipped with a fireplace and the building could be quite easily reassembled and relocated. After the second world war, Finland quickly started the reconstruction of the country, and later in the 1970s, industrialized element-based house building was a way to build efficiently. At that time, flexibility was more related to the ability to reassemble the elements. Since the 1980s, flexibility in office buildings has advanced as some big building owners, such as Senate Properties (the owner of the Finnish real estate assets in Finland), have demanded it. However, between 1990 and 2010, flexible solutions in residential buildings only appeared through some advanced builders. (c.f. Hakaste, 2014; Tarpio, 2015; Tarpio et al., 2021) Research on the current drivers and market solutions for flexible residential housing in Finland is scarce.

Thus, this article investigates the needs and solutions for flexible housing in Finland. The aim is to understand the preparedness of the markets to provide flexible housing solutions and the willingness of the markets toward flexibility. More specifically, the study seeks to answer the following research questions reflecting three main perspectives, namely, (1) housing trends, (2) stakeholders’ views and (3) market solutions:

- RQ1: Which housing trends lead to flexibility? Are there patents for flexible housing solutions?
- RQ2: How is housing flexibility viewed?
- RQ3: What kinds of market solutions exist for flexible housing? Do they include sustainability-related aspects?

The remaining sections of the paper are organized as follows: Sect. 2 describes the background of flexible housing, including the different hierarchical flexibility levels, Sect. 3 introduces the research methodology used, Sect. 4 presents the research results and discusses the findings, and Sect. 5 concludes the study.

## Background on flexible housing

Functional adaptability and multifunctionality are closely related to flexibility. Functional adaptability, also referred to as transformability, can be achieved through structural or technical changes. ISO 20887:2020 standard (ISO 20887:2020, 2020) provides three design principles of housing flexibility: versatility, convertibility, and expandability. De Paris and Lopes (2018) list various strategies for designing flexible houses, such as service and technical areas, easy access to maintenance, separation into permanent and impermanent areas, and application of movable partitions and furniture. Flexibility can also be considered through functional and customer requirements (Malakouti et al., 2019). As proposed by the framework of Lans and Hofland (2005), functional requirements can be, for example, neutral for furnishing, the possibility for a change of floor plan, the possibility to reshape apartments, modernization flexibility, character flexibility, flexibility for changing safety requirements, wheelchair adaptability, capacity for expansion, multifunctionality, financing flexibility, capacity to shrink, parking flexibility and robustness for calamities. Malakouti et al. (2019) assessed how these alternatives meet the criteria set for customer requirements related to location, site, unit, and performance in use. Based on these previous definitions, the authors consider flexibility as an umbrella term for various concepts that provide flexibility in practice, such as adaptability, convertability, versatility, multifunctionality, modularity, and transferability. The authors define adaptability as the ability to structurally change or modify the space to suit a particular use. In contrast, versatility is the ability to adapt to different functions or purposes with small changes to the space.

**Fig. 2:**
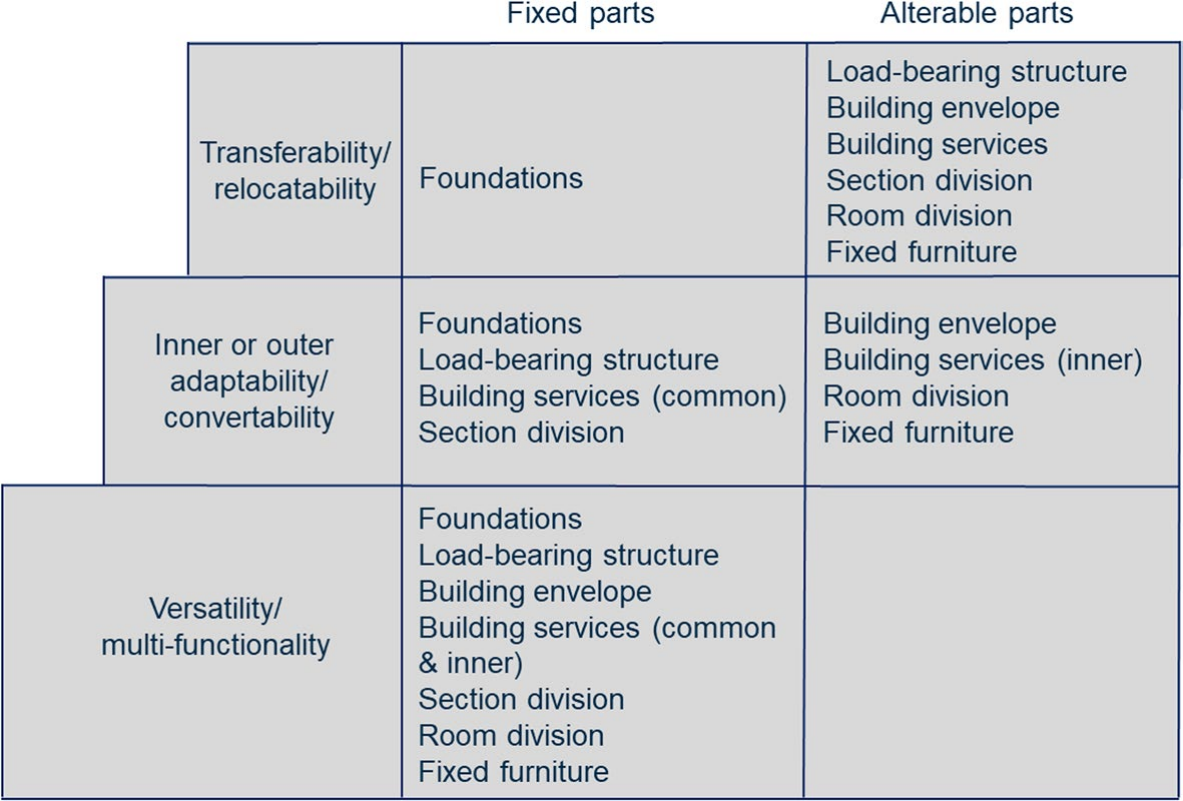
The fixed and alterable parts of a house on different hierarchical flexibility levels (modified from Tarpio’s table, p. 70 (Tarpio, 2021))

Tarpio (2021) notes that from the circularity point of view, it is more convenient to consider flexibility as hierarchical levels, where the lowest level (versatility, multifunctionality) is the lowest-cost solution and requires the least amount of changes. In contrast, the top-level (transferability) is usually the most expensive solution (Fig. 2). Furthermore, Tarpio (2021) sees that multifunctionality occurs when only the movable furniture’s location is changed, whereas adaptability necessitates either inner or outer structural changes.

Modular design can increase building adaptability and reduce the cost of construction (Ling et al., 2021). Modular architectural designs are expected to enable better maintainability and adaptability and be cost-competitive (Ling et al., 2021). Typically, modular buildings refer to buildings with prefabricated parts or components (Ferdous et al., 2019). In general, however, parts or rooms in such buildings cannot be removed and moved to a new location once they have been assembled, but relocatable modular buildings also exist, e.g., Kyrö et al. (2019). However, it must be remembered that the construction of new buildings from nonrenewable materials is not the best solution in terms of sustainability. New and renovation construction must be possible to meet the current and changing needs with fewer materials. For example, from a circular economy perspective, the reuse of materials is possible in housing solutions. However, well-established and standardized practices for their utilization are lacking (e.g., Benachio et al., 2020).

Even if Machado and Morioka (2021) studied the contributions of modularity to the circular economy from a generic perspective, their conclusion on the importance of the link between modularity and the circular economy is also relevant in the construction sector. The design phase of the building is critical for sustainability since the building materials and energy system are chosen, and decisions are made that affect the maintainability. These decisions can either increase or decrease the building's carbon footprint. Building designs that are based on flexibility and adaptability are sustainable as they can meet the changing living needs and preserve the value of the building, thus allowing for longer usage of the building (Estaji, 2017). Flexible designs allow users to control and change their own space, and research shows that users want to enlarge, narrow, divide and combine their space (Özinal & Erman, 2021). Schneider and Till (2005) have even argued that for the house to be environmentally, socially, and economically viable, its design has to include flexibility.

The EU Level(s) framework, primarily developed for measuring the sustainability of residential buildings or offices, also has an indicator for flexibility. The framework consists of six macro-objectives. The second macro-objective, ‘Resource-efficient and circular material life cycles’, aims to optimize the building design to support lean and circular flows, such as flexibility to adapt to change. Each macro-objective has two to four indicators. For example, the second macro-objective has four indicators, of which one—2.3 Design for adaptability and renovation—examines flexibility in housing design. The unit of measurement is the ‘adaptability score’. Another indicator examining adaptability and deconstruction is ‘2.4 Design for deconstruction, reuse and recycling’. Its unit of measurement is the deconstruction score (Dodd et al., 2021; European Commission, 2021b).

German Sustainable Building Council (DGNB) has developed the ECO2.1 Flexibility and adaptability with seven technical indicators—space efficiency, ceiling height, depth of floor plan, vertical access, floor layout, structure, and building services—for housing flexibility. According to DGNB, building adaptability can reduce costs and extend building service life while increasing user satisfaction (DGNB German Sustainable Building Council, 2021).

## Research methodology

**Fig. 3:**
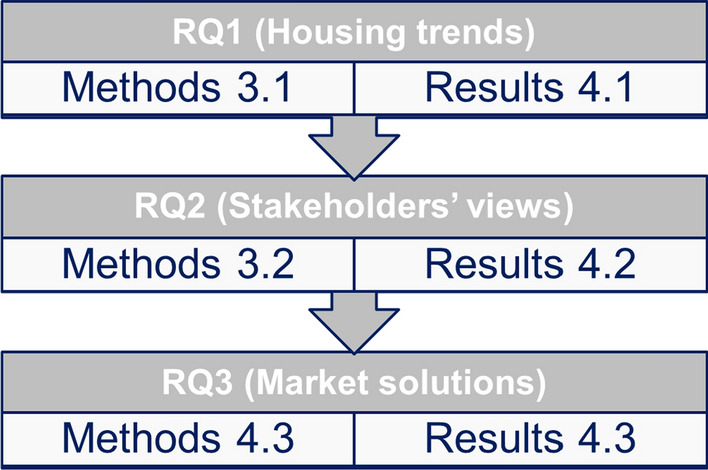
The research approach

We selected an exploratory approach to understand flexibility in housing, especially the trends and benefits of flexible housing, but also the challenges, market demand, and solutions for flexible housing. We collected data by conducting a trend search, including market examples and patents, stakeholder interviews, and market analysis. The actual study proceeded as described in the following Sects. 3.1–3.3. Correspondingly, the results are described and discussed in Sects. 4.1–4.3. Figure 3 outlines this approach.

### Searches for trends and patents

We searched for housing-related trends from a flexibility viewpoint. We accepted a trend only if we found a reference dealing with trends from a few recent years, i.e., we did not ‘invent’ any trend but collected trends from trends-related literature. In addition, we sought at least one market example for each accepted trend. If no market examples were found, an issue was not considered a housing-related trend.

We searched the relevant patent families using the STN® information retrieval tool (FIZ Karlsruhe GmbH, 2021a) with the WPINDEX database (FIZ Karlsruhe GmbH, 2021b), providing information on patent publications from the world's 52 most important patent issuing authorities. We carried out searches from the year 2000 onwards. 

Table 1 describes the performed patent searches.

**Table 1:** The patent searches

Search	Number of patent families (*indicates that the results were relevant and used in the analysis)
“Relocatable building”	29*
“Expandable house”	16*
“Modular building” + E04B/ipc,cpc (E04B patent classification: building)	1400
The previous + “multifunctional? or adaptability or reuseability or recyclability or transferability”	20*
Flexible housing	Nothing relevant found
Multifunctional? or adaptability or easy maintenance or deconstruction or service life? or life cycle analysis or reuseability or recyclability or transferability + E04B/ipc,cpc -transforma?	160, not interesting
The previous -flexible modular? hous? or flexibility during use	2 frames for easily moveable relocatable house

### Stakeholder interviews

**Fig. 4:**
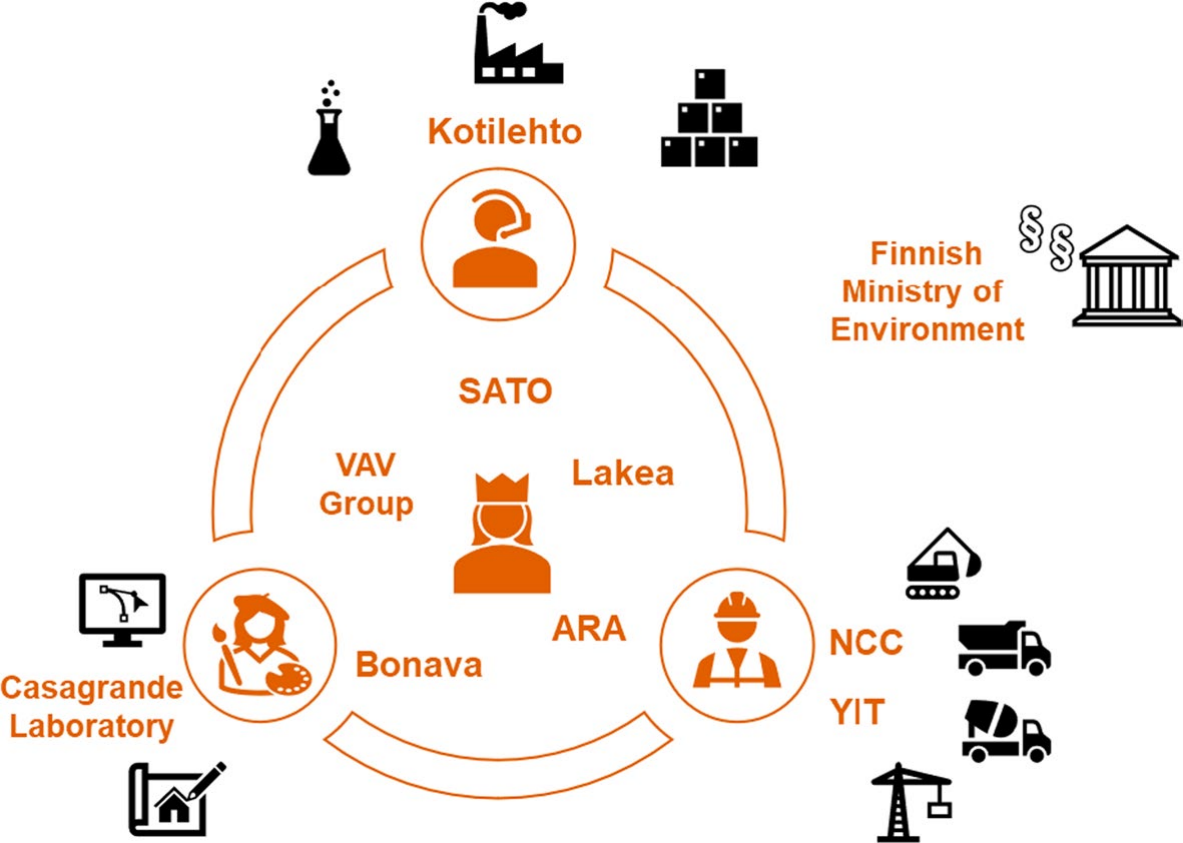
The value chains are connected to a value network that serves the customers

We conducted 12 structured stakeholder interviews in Finland to explore (1) flexibility and modularity in housing and their advantages, (2) the challenges in increasing flexibility in housing, and (3) the readiness of housing markets to produce flexible modular housing solutions. The detailed interview questions are presented in the “Appendix”. The interviews were conducted in November and December 2021, and each interview lasted between 33 and 63 min. They were not recorded, but we took detailed notes during the interviews. All interviewees were well-experienced experts, most of them with at least 20 years of work experience. Table 2 provides basic information on the interviewees. We interviewed representatives from the value chain (except dwellers), including construction companies, designers, housing providers, financers and regulatory authorities (Fig. 4).

**Table 2:** Description of the interviewees

Organization (name)	Interviewee’s organizational role	Years of working experience	Interview date	Interview length (min)
Construction company (NCC Oy)	Industry director	32	11.11.2021	33
	Design manager	24	18.11.2021	38
The Housing Finance and Development Centre of Finland (ARA)	Development manager	39	11.11.2021	63
Building regulations authority (Finnish Ministry of the Environment)	Senior advisor/specialist	24	16.11.2021	30
Architecture and innovation company (Casagrande Laboratory)	Principal Architect	20	22.11.2021	50
A company offering rental apartments in various cities (SATO Oy)	Vice president, investments	29	23.11.2021	49
Urban developer and construction company (YIT)	Vice president, Strategy and Development	30	25.11.2021	57
A company—building and owning rental apartments—owned by the city of Vantaa (VAV Group)	CEO	20	26.11.2021	36
A real estate agency in Vantaa (Lakea Oy)	Business director, Construction and Property Management	35	29.11.2021	40
A residential development company, developing and selling homes in Northern Europe (Bonava Oy)	Chief designer and commercial director	25	7.12.2021	45
A company designing and constructing CLT prefabricated cottages (Kotilehto Oy)	CEO, sales, design, and construction	12	14.12.2021	45

We used a purposive sampling logic to select the interviewees (Patton, 2002). We selected interviewees who were very experienced in their fields and had worked for years in the fields of modularity, flexibility, and sustainability. The design and construction sector is fragmented into value chains that connect customers (building owners and tenants), designers, the building material industry, construction companies, and the Ministry of Environment. Therefore, we wanted to interview various stakeholders in the housing value chains. Figure 4 illustrates the place of the interviewees in the value chains. However, some stakeholders may represent two functions, so the figure is merely indicative.

The interview analysis process was iterative between previous research articles and our empirical data. We first read through the interview notes and analyzed them together to understand their meaning. Then, we went back to the identified previous research articles to understand the novelty of our findings.

### Search for market solutions and assessment of their sustainability

The authors collected examples of market solutions that represent housing flexibility and consider sustainability in housing. Their sustainability was roughly assessed qualitatively from all three core aspects: social, economic, and environmental. The analysis was mainly based on the market solution providers’ websites, and if the information was not available, the authors analyzed the solution themselves.

## Results and discussion

The results section is divided into three main parts. First, we present trends supporting housing flexibility. Then, we provide findings on stakeholder views on flexible housing, covering meaning, benefits, challenges, market demand, and technical solutions for flexibility. Finally, we discuss market solutions for flexible housing.

### Trends and patents supporting housing flexibility

#### Housing-related trends

Table 3 lists housing trends promoting flexibility and market solutions responding to these trends. Some of the market solutions are extreme, but they are still showing which kinds of market solutions were discovered. Although flexibility per se does not exist as an independent housing trend, many housing trends support flexible housing solutions. This is confirmed by the fact that market solutions or at least pilot implementations were found for each trend. It should also be noted that the mentioned trends also promote other market needs but here, only flexibility-related aspects are raised. Future will show if the solutions find a place in the wider market.

**Table 3:** Flexibility-oriented housing trends and examples of corresponding market solutions

Housing-related trend	Market examples
Extending the service lifetime of buildings and materials efficiency (IEA, 2021)	Pyörre-house (Aulis Lundell Oy, 2021) is a low-carbon circular economy model house, designed according to the EU’s design criteria for versatility, dismantling, and recyclability. More than 80 percent of the materials (e.g., steel) can be reused or recovered. The carbon footprint is less than 15 kgCO_2_e/m^2^ and, thanks to the recyclability of steel structures, the carbon footprint rises to almost −12 kgCO_2_e/m^2^. The house stands firmly on steel legs (stop digging ground screw); the soil has not had to be destroyed. Environmentally-friendly solutions, such as air–water heat pumps and solar panels, have also been utilized in energy production. Energy efficiency class is A. Carbon sinks are created in the yard with the help of plants, biocarics, and recycled concrete crush
Remote working, work-at-home, living/space-as-a-service (Global Proptech, 2021)	VALO Hotel Helsinki (VALO Hotel & Work Helsinki, 2021) allows both working and sleeping in a single room. The bed can be easily transformed into a sofa Noli hotel (NREP, 2021) allows both short and long-term visits and is built on the idea of supporting communality. The smallest room in the hotel is 15 m^2^ in size, and the largest is 38 m^2^. Each room has a balcony, a well-equipped kitchen, and a washing machine in the bathroom
Downsizing of physical belongings and anti-consumption (Winther, 2021)	Jetta-minitalo selection includes several small detached houses that can be classified as mini-houses (Jetta-talo, 2021). The smallest model has a living area of 49 m^2^. Despite their small size, the mini-houses have all the facilities and functions needed for today’s living
Concerns about housing affordability and the high price of land (PwC & the Urban Land Institute, 2022)	Nestinbox (Moderna Trähus (Modern Woodenhouses) and the architects Elisabetta Gabrielli and Pontus Öhman, 2021) resembles a birdhouse that is attached to a rock wall, a concept of building on vertical ground. The size is 48.5 m^2^. Allows people to build on vertical rock walls, no need for expensive land. The concept is now in the commercialization phase. Target group: adults. Smart and resource-efficient wooden (CLT) house that does not require land to build on, “carbon dioxide-free foundation work”
Co-living (ARUP & Ellen MacArthur Foundation, 2020)	Kotikatu 365 (Corgroup, 2021) is a concept where inhabitants are offered a modern dwelling, common spaces with activities, equipment, and vehicles for shared use, and services that ease everyday life in cities
Circular economy in cities (BNP Paribas Real Estate, 2021; Deloitte, 2021)	Circulariteit (Van Wijnen Groep, 2021) by Van Wijnen provides houses that can be disassembled and moved to another location. The pop-up neighborhood lives self-sufficiently. Circularity is a key concept in the neighborhood, innovations in housing, mobility, energy, and materials Peikko (Peikko Group, 2021; Peikko Group Global, 2021) offers bolted connections for concrete buildings providing the buildings with a possibility to be relocated. Bolted connections increase the reusability of precast concrete structures without compromising load‑bearing capacity. Furthermore, removable structures could also be replaced if they are damaged or deteriorated
Urbanization (OECD, 2020)	Minitopia (Eindhoven News, 2021) is a concept that allows future residents to design and build their home buildings (max 100 m^2^). Empowers people to design their mini home with a low carbon footprint. The North Brabant province financially (500 k€) supports the initiative. The Municipality of Eindhoven is making the land available and is preparing it for construction and residential use. The land can be rented. Target group: self-builders. With sustainable living forms that are compact, flexible, ready-made or modular. Designed with an app and built with natural building materials or by making smart use of existing materials
Digitalization (BNP Paribas Real Estate, 2021)	Munger Hall (The Guardian | Architecture, 2021; UC Santa Barbara, 2021) is a proposed dormitory at the University of California, Santa Barbara, for 4500 students in an 11-story building whose bedrooms and common areas are mostly devoid of windows. Instead of windows, rooms will have glowing screens that mimic sunlight
Living alone (Ruuskanen et al., 2019; Statistics Finland, 2021b), the number of single-person households is growing (Tervo & Hirvonen, 2020)	SATO’s new studios (Hammarsten, 2019) are no longer bounded by load-bearing walls, thus allowing for flexibility. In a couple of years, all new studios of SATO houses will be flexible and can be combined with neighboring apartments. SATO will make lightweight partitions that can be removed or a door made into them The demand for studio apartments is high in Finland, so SATO is offering small (15 m^2^) dwellings, called studio homes, that offer the residents the option to interact with their neighbours (SATO, 2021)

Furthermore, policy means drive toward the listed trends. Finland has an ambitious national goal of achieving carbon neutrality by 2035 (Finnish Government, 2019), even if the general goal in the EU is to reach this level by 2050 (European Commission, 2020a). According to the Finnish sector-specific low-carbon roadmaps (Paloneva & Takamäki, 2021), Finland's property and construction business can significantly reduce emissions by 2025 through more efficient utilization of resources and space as the circular economy. Furthermore, to inform and support actors along the construction value chain, the European Commission has launched principles for circular design of buildings (European Commission, 2020b). Flexible housing solutions contribute to this development.

In the US context (Mutter, 2013), the trend of downscaling in home size is explained by motivations including interest in a simpler life, sustainability and environmentalism, cost, freedom and mobility, a sense of community, and an interest in design. It can be assumed that similar motivations also exist elsewhere. Therefore, the downscaling of home size will be critical for pursuing a sustainable consumption transition (Cohen, 2021).

Affordable housing is an issue of concern in Finland, especially in the capital area, because housing is expensive (Housing Europe, 2021). Both the rents and the prices of houses and apartments are more expensive than in the other parts of the country. This is partly caused by internal migration due to better employment opportunities (Housing Europe, 2021). On the other hand, those living in the Helsinki region are more concerned about climate change than those living elsewhere (YIT, 2021), so the environmental aspects are more emphasized in housing.

Also, in US cities like New York and San Francisco, the share of single-person households is growing, leading to micro-apartments offerings (Cohen, 2021). In Finland, such an offering exists (SATO, 2021), although it has been criticized (YLE, 2021a). Still, there are queues for micro-apartments in the Helsinki region (YLE, 2021b).

#### Patent families

**Fig. 5:**
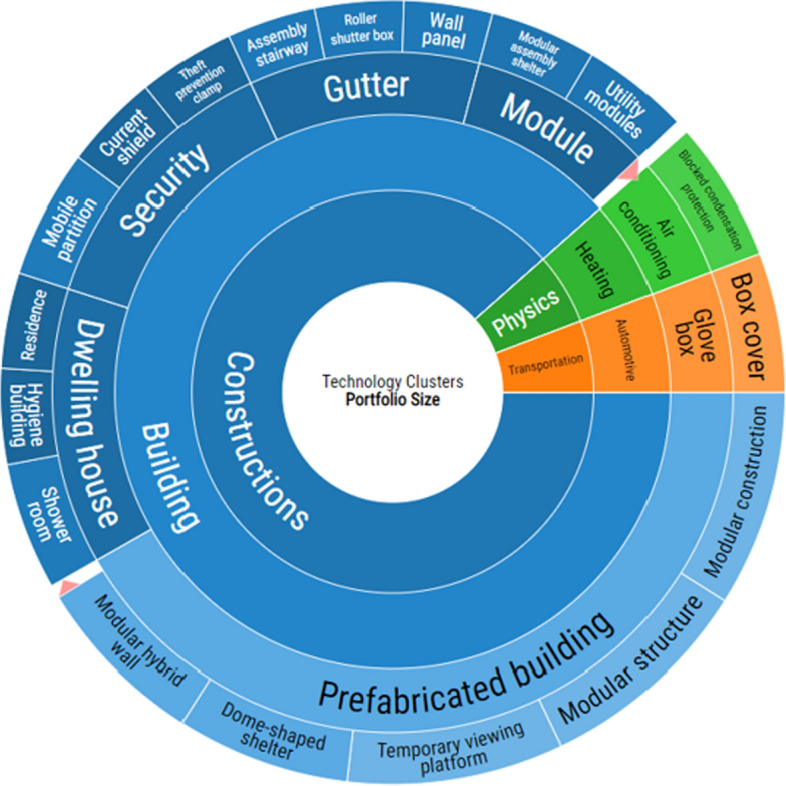
Technology clusters (The analysis is made using LexisNexis PatentSight (LexisNexis, n.d.).)

Figure 5 shows the analysis of the patent families divided into technology clusters. Forty-six patent families were found interesting for this research. In the figure, the width of the sector refers to the number of patents found. Most of the patents are related to constructions but a few also to physics and transportation. All constructions-related patents are related to buildings, the most common subarea being ‘prefabricated building’.

**Fig. 6:**
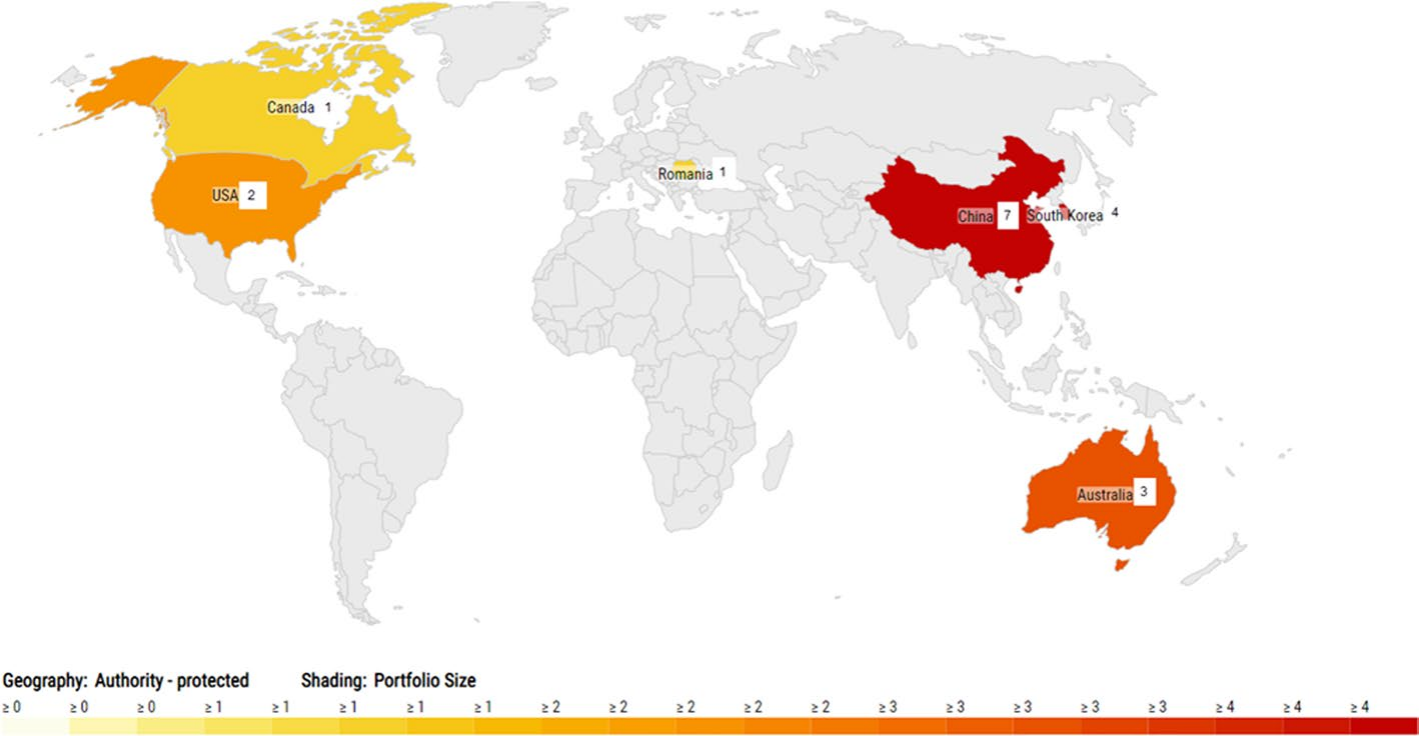
Geographical distribution of the active patents (The analysis is made using LexisNexis PatentSight (LexisNexis, n.d.).)

Figure 6 shows how the active patents are geographically distributed. Seventeen active patent families can be identified within the 46 patent families. Active means that they are in force or pending. It is worth noting that Europe's role in active patents is almost non-existent. During 2006–2010, there were 759 building-related patent applications only in Finland (Bröchner, 2013). Compared to this, the 46 found patents families globally since 2000 seems to be a very low number. Development work in this area does not appear to be very active. This may also emphasize the low interest of the construction industry in flexible housing.

### Views on flexible housing

#### Meaning of flexible housing

The interviewees generally agree that flexibility in housing means the ability to use the facility according to their current needs. This is in accordance with previous definitions (Cellucci & Di Sivo, 2015; Estaji, 2017; Zairul, 2021). For example, Zairul (p. 8) has defined flexibility as “the ability to change the usability according to changing needs” (Zairul, 2021). The interviewed construction companies and most building owners talked about apartment buildings. According to these interviewees, flexibility in apartment buildings means either multifunctionality inside a dwelling (e.g., using flexible furniture) or the ability to unite or separate two dwellings structurally. Then again, interpreting other interviewees, housing flexibility in small houses refers mainly to transferability and multifunctionality. Thus, multifunctionality is a common denominator for apartment buildings and small houses. This finding is perhaps not surprising as previous research has found that multifunctionality and extendibility of spaces are the most influential flexibility components of residential buildings, increasing the housing quality (Malakouti et al., 2019).

Two interviewees—with architectural backgrounds—mentioned that flexibility has been at the core of living and house design since humans started to build houses. Several types of systems and solutions have been designed to allow several people to sleep under the same roof or share a sauna. At that time, “communality” was the typical way of living. After that period, people were “compartmentalized” into concrete apartments. Nowadays, the trend is again towards sustainability and “communality”, and flexibility is one way to support them. These views support earlier descriptions of flexibility in the history of residential buildings (c.f. Hakaste, 2014; Tarpio, 2015; Tarpio et al., 2021).

Currently, criteria for flexible housing are being developed by the EU Levels (European Commission, 2021b). The existing criteria are mainly for offices (Levels) or apartment houses (DGNB ECO2.1 (DGNB German Sustainable Building Council, 2021)). Some issues are easy to quantify, but others are not that easy, such as the easiness of opening joints.

Two interviewees said that flexible housing planning needs to start with zoning. For example, the flexibility of an individual real estate to adapt to different uses during its life cycle depends on the limits allowed by zoning. This is confirmed by Somersalmi, who states that zoning that allows the building of various real estate types also enables the use of the space as a service concept in the circular economy (Somersalmi, 2021). Zones get outdated in 10 years. If the zone is outdated, construction costs increase when the builders and owners need to wait for the updates. The interviewees were wondering whether flexible zoning could exist. Flexibility can also mean the construction of a new floor above an existing apartment building. In this case, the new floor is often built as wooden modules and lifted above the existing floors.

Based on the construction company’s customer feedback, the apartment must be well-designed and built, but the location is also important. Floor plans will come third in the customers’ wishes. Today, the customer can view the future home in a 3D model, which helps because reading 2D drawings is not easy for everyone. People appreciate the opportunity for change, but making an apartment they dream of during construction is important, as people do not think very far into the future. Then, if necessary, people move to a new apartment that better meets their needs.

#### Benefits of flexible housing

There are several benefits flexible housing could provide. First, it could offer the possibility to increase or decrease housing space when needed. Then, housing would adapt better to different life situations and support people living in their homes during different life phases. For example, many marriages end up with a divorce in Finland, but often the parents want to stay close to each other to ease the everyday living of two families. On the other hand, flexible housing solutions could unite different generations if two apartments can be united or separated depending on the situation. Furthermore, especially group building investors consider the possibility of decreasing the size of the apartment later on, when kids have moved out, to remain in the community. Furthermore, flexible housing supports sudden unexpected space requirements such as remote working needs during Covid-19.

One interviewed company stated that flexible housing solutions widen their product offering. Therefore, they have been developing a related solution and want to continue to offer this option. When designing housing flexibility, certain issues need to be locked up early during the planning process. However, housing flexibility also allows the owner to move certain decisions to a later stage. For example, it may allow transforming a closet into a sauna later on if the layout is designed to support it.

Modular construction can facilitate housing flexibility when the building is transferable. For example, municipalities are already using temporary modular schools that can be transferred to a new location when needed. The reason for their usage is often the quickly changing demographics (Kyrö et al., 2019). These modular, transferable schools can also be considered sustainable as they are made from wood, and industrial production optimizes materials usage (Parmaco, 2021). Furthermore, Kyrö et al. found that these relocatable modular buildings support circularity (Kyrö et al., 2019). In addition, industrial modular construction can shorten the onsite construction time.

#### Challenges for flexible housing

The older apartment buildings do not meet the current need for smaller apartments. Also, structural changes are difficult in older buildings, as they have not been designed for flexibility. For example, the location of sewer and water points should be changeable, but in practice, it would mean high renovation costs. The older buildings are modernized during energy-efficiency renovations. Transforming a rental apartment to an office building can be costly since the floor height in apartments is standardized and lower than in commercial or office buildings. Lightweight partitions are hardly a cost issue.

Lifecycle housing supports the housing of the elderly when a house can be furnished so that a person can move with a rollator. However, these solutions are usually expensive; only wealthy people can afford them. Sometimes, service homes for the elderly are built as dwellings, in which case, they can be transformed into normal dwelling use.

A bigger apartment (75 m^2^) can be designed to be divided into two departments later on. However, this increases the design costs as two layouts need to be designed—for the bigger apartment to be built and the other two possible smaller apartments. Since designing and constructing apartment buildings with dwellings where a studio can be separated requires double work in the design and construction phases, these dwellings cost more than traditional dwellings without flexibility.

Often, the builders do not invest enough in the design and construction costs. Therefore, the research needed to build housing flexibility is not done properly, which means that the cost of the needed changes later on will be higher than it would have been if the design had been done thoroughly. One hindrance to housing flexibility is the challenge of moving building services. Also, Tarpio et al. (2021) refer to the lack of solutions for building services that support internal transformability. The owners do not want the inhabitants to adjust the building services to mess with the systems. Flexibility between apartments necessitates ensuring that the building services systems are separate or have provisions for kitchens, bathrooms, and restrooms if the dwellings are separated later. In addition, cold and hot water measurements must be possible in both apartments. Furthermore, fire compartmentation and sound insulation are hard to design to be flexible. One company has prepared separate design principles for designing flexible dwellings. These principles are meant to tackle all possible technical challenges that may occur due to designing a dwelling that can be separated into two individual dwellings.

The housing factories are reluctant to change their operations during a boom and cannot afford to make developments during a downturn. As a result, the development of flexible housing solutions is sluggish or non-existent. If the situation were to change, some support for development work would be needed.

#### Market demand for flexible housing

According to the interviewees with Finnish stakeholders, the market demand for flexible apartment buildings is very low. However, it can be considered as a limitation that the potential dwellers were not interviewed. So, the conclusion is based on other stakeholders’ views. But the interviewed stakeholders included all other stakeholders in the value chain (see Fig. 4). People tend to choose a cheaper apartment if they need to choose between an apartment with the possibility of flexibility and an apartment with no flexibility. Furthermore, people do not plan their living in the long term; instead, they tend to move to another apartment that better suits their needs. Overall, academic studies of dwellers’ wishes are lacking. But a Turkish study found that users demand flexible housing, which enables their spaces to be enlarged or narrowed (Özinal & Erman, 2021), which contradicts our Finnish interviews. In Vartia’s (2015) study, one couple that had bought a dwelling with the option to separate a unit from the dwelling told they might do the separation if they lived abroad during the winters.

Construction companies that build for professional investors see no incentives for flexibility and adaptability in housing. On the other hand, long-term owners that build into their own balance are interested in flexibility and adaptability since they increase the service life of the building and, in the best case, reduce maintenance costs if building parts can be easily replaced, referring to flexibility in design.

The owners of buildings need to demand flexibility and adaptability; otherwise, these concepts are not part of the house design or implemented during construction. According to Tarpio et al. (2021), architects conceived housing developers’ disinterest in including adaptability in their projects as the primary obstacle. However, their interviews did not include any other designers but architects. Changing the house afterwards is expensive since the electricity, ventilation ducts, and water pipes might need to be transferred to another place, necessitating design and demolition. However, it is possible to include flexibility in the house during the design, but it must consider all aspects, not only structures but also building services. Currently, the trend is to build small homes because the market will buy them, which benefits investors, especially investment funds. In addition, the zone and authorities’ requirements need to be considered when designing, as they may restrict certain types of houses. For example, a three-room apartment may be required to be a certain size in square meters.

Also, marketing these flexible dwellings necessitates extra work, as people do not yet understand what these kinds of dwellings mean in practice. If there were more talk about flexible housing solutions, for example, in the media, their awareness would increase, and their demand could also increase. The marketing of the dwellings should also be clear and concise, explaining the options more understandably.

A recently conducted Finnish housing preference study does mention the demand for flexibility. But instead, dissatisfaction with current housing is mainly caused by lack of space (especially among young adults), too high housing costs, longing for their own yard, and desire to live closer to nature (Omakotiliitto, 2020). The survey received 1556 answers in 2019, and it discovered that the most wanted housing form is a detached house, especially among young families in Finland, but 68% consider that the housing prices are too high (Omakotiliitto, 2020).

Another survey confirms that people want more space, and many respondents said they dream of a home of 76–130 m^2^ (Lähitapiola, 2020). Practicality and functionality are the most important features associated with a dream home (Lähitapiola, 2020). The survey confirms that Finns want to live in a detached house and own it. In addition to being close to nature, the dream home is expected to be peaceful and well connected. In terms of equipment, own sauna, yard or garden are often wanted. A balcony or terrace, storage space, and a spacious and functional kitchen are also appreciated. The survey received 1068 answers in 2020 (Lähitapiola, 2020).

#### Technical solutions providing flexibility

Different kinds of market solutions exist. Nowadays, many of the interviewed housing providers design for flexibility, using slight partitions or having openings in reinforced concrete walls to add a door if necessary. Some small dwellings include adaptability by moving walls or sliding partitions to divide spaces to use all the precious space. Also, smart, movable storage solutions and fold-out furniture, such as fold-out beds, are used in small spaces. The room can be used for dining, working, relaxing, or sleeping in extremely adaptable one-room dwellings. For example, the Domestic Transformer (EDGE Design Institute Ltd, 2016) represents adaptable housing. Similar solutions and reconfigurable layouts are represented by the architecture and design magazine Dezeen (2021). Some households have even bought a small house for their yard for remote work during Covid-19 (Sivonen, 2021).

Apartment buildings can include flexibility either inside one apartment or between two apartments. Flexibility inside an apartment means using multifunctional furniture that can serve two purposes: a sofa during the day and a bed during the night (BoConcept, 2021; LINAK Oy, 2021). Flexibility between two apartments necessitates structural changes. One solution is separating a side apartment (Bonava Oy, 2021), which requires closing the doorway between the apartments and installing a kitchen in the side apartment. The side apartment has a reservation for drains and water pipes for the kitchen. The doorway between the apartments will be closed to meet the fire and sound insulation requirements between the apartments. Both apartments have their own ventilation systems and separate electricity and water consumption measurement systems. In addition, both apartments have their own warehouses. However, the demand and response might not meet at the right time, as it is not usually feasible to buy or sell the apartments next to you when needed.

In Finland, the apartment is owned by a special form of a limited liability company where the shareholders are co-owners. This legal form of a housing company ensures that the shareholders need to pay all monthly payments as long as they are shareholders, thus preventing the bankruptcy of the apartment building company (Lujanen, 2010). Hence, in Finnish housing companies, flexibility between two apartments (either uniting or separating them) necessitates creating two different stock series. If two apartments are united, an amendment will be made to the Articles of Association, which will require a decision by the Board of Directors of the housing association. The articles of association should allow for the parallel holding of two series of shares (Asunto-Osakeyhtiölaki 1599/2009 (Housing Company Act, in Finnish), 2009). In rental housing, similar arrangements are not needed.

Many of the interviewees mentioned a modular bathroom pod as one example of a solution that saves construction time and reduces construction costs. The pod is industrially manufactured, so it can be considered a sustainable way of construction as material usage can be optimized and waste is reduced. Also, the construction conditions are more optimal as the humidity levels can be controlled, whereas onsite construction necessitates air dryers that consume energy. However, the markets lack simple modular solutions that could be easily exported abroad to places with an urgent need for space. The modules would need to meet the structural and energy efficiency needs. Many of the current modular solutions are too complex for transportation abroad.

Solid wood elements allow flexibility differently from bricks or concrete since wood bends, and a door (a hole) can be more easily made to a wooden wall than a concrete/brick wall. Even if cross-laminated timber (CLT) also offers other benefits (Waugh Thistleton Architects, 2018) but flexibility, there is some evidence that the construction costs may be higher when utilizing CLT-based solutions (Ahmed & Arocho, 2021). Furthermore, the Finnish Building Codes allow solid wood buildings to deviate from general energy efficiency requirements (Ympäristöministeriön Asetus Uuden Rakennuksen Energiatehokkuudesta 1010/2017 (Finnish Ministry of the Environment on the Energy Efficiency of a New Building, in Finnish), 2017). As a result, the energy efficiency of solid wood buildings is often lower than that of other buildings in Finland.

Transferable market solutions are usually designed for nursing homes and schools, less for houses. Still, several builders provide relocatable leisure homes in Finland that could even be enlarged into bigger modular homes.

### Market solutions

Market solutions focus on Finland, but some solutions are also highlighted elsewhere. Even if construction regulations and energy demands vary from country to country, international examples can inspire the development of housing solutions suitable for certain climatic conditions. In Table 4, the identified market solutions are classified as being either ‘Small, transferable houses built in a factory and assembled at the site or the factory’ or ‘Apartments allowing either multifunctionality or uniting or separating two apartments’.

**Table 4:** Identified flexible housing solutions and rough assessment of their sustainability

Housing market solution	Social	Economic	Environmental
*Small, transferable houses built in a factory and assembled at the site or the factory*			
*Olokoto* (OLOKOTO Oy 2020) is built of log in a factory, and high-quality materials (top-level insulation) are used. A hydraulic foundation, electric systems, electric heating, ventilation, and plumbing systems	Meets changing space needs, aesthetic, designed for nature experiences	Expensive, but the calculated life cycle of the log frame can be 100 years. Quickly in use in ten weeks Target group: wealthy adults	A log is an ecologically sustainable building material. The insulation of Olokoto is comparable to the insulation of a modern single-family house
*ProModi* (Lundell Oy, 2021) is built of modules that can be added, subtracted, separated, or moved. The modules are built in a factory. Interior indoors slide inside the steel frame wall	Meets changing space needs	Webpages state that the building is competitively priced. Due to the lightness of the modules, large groundworks for foundations are not required Target group: wealthy adults	Solar power is utilized. The light and recyclable building material and flexibility support the ecology and longevity of the house. At the end of the life cycle, the module frame is 95% recycled or reused only by renewing the surfaces; thus, the module is basically eternal
*Domino Homes* (DOMINO HOMES, 2021) are modular, prefabricated container homes, which are made from shipping containers combined with timber frame construction	The containers appear modern	No mention of the cost Target groups: adults	Containers are eco-friendly and zero-energy ready, as they are repurposed into homes instead of being melted down. In addition, natural materials are used in the construction process
*WikiHouseNL* (WikiHouse NL, 2021) is based on a digital, open source, do-it-yourself concept. The building is built using computer numerical control (CNC) machines and assembled at the site. The building can be reassembled	The do-it-yourself concept empowers people	The digital concepts are free Taking the concept as a baseline and building oneself, one can save up to about 25% on the construction costs	The house is resource-efficient due to precisely digitally produced parts and energy-efficient due to high-level insulation
*Tumbleweed Tiny Houses* (Tumbleweed Tiny House Company, 2021) are small houses that can be towed by a car. The design of the home takes 10 min using predesigned floorplans	Easy to design and buy online. Easily movable with a car big enough	The cost of one tiny house is approximately 100 k$. The tiny house can be bought with the help of a Tiny House Loan	The tiny house is certified GREEN by TRA Certifications
*Bokompakt Student Housing* (AF Bostäder, 2014) represents compact living for students	The concept meets student housing needs	Target group: student	Resource-efficient. The energy use (heating, hot water, and building electricity) is ~ 500 kWh/resident/year—solar panels in use. Depending on the weather, the building is self-sufficient in electricity
*Minihouse* (Minitalo Oy, 2021) is a small home, which is fast delivered through a turnkey concept	The buyer can order the whole building project; no know-how is needed. A solution to be put in one’s backyard	The cost of the mini house varies between 100 and 200 k€ Target group: young adults	Resource-efficient and makes more efficient use of the plot of a detached house that has become unnecessarily large and requires maintenance
*CLT prefabricated cottage* (Kotilehto, 2021) is prefabricated in a factory and transported as a ready cottage to the site	The customer can easily buy a cottage and have it transferred to the site as a ready package	No mention of the cost. An offer needs to be asked for	The cottage is made of CLT; no waste is basically generated as the by-products can be utilized 100% both as crumbs and by further processing. The wood used in the manufacture of the CLT board is Finnish PEFC-certified softwood
*KONTU transferable mini house* (Carpe Team Oy, 2021) can be extended and transferred easily. It can be a mini-home, a holiday home, a sauna building, or even a mini-office, and it is brought ready-made on site	The house empowers the resident to increase the size of their current home with additional space	Ready to move in from 19 to 79 k€, depending on the house size. The price does not include connections and other site work, foundations, transport, water meter and gutters	The house is made of non-insulated CLT since the Finnish Building Codes do not require the insulation of solid wood structures. However, this reduces the energy efficiency of the building
*CHP Village* (CPH Rakennusmaailma, 2021; Village, 2021) provides Danish student buildings that are designed out of shipping containers	Supports the creation of sustainable villages and targets to curb the shortage of student accommodation	Living in a student apartment costs about 600 €/month Target group: students	Reusing shipping containers reduces the use of virgin materials for building new houses and can be considered a sustainable action. The interiors are made from wood
*Easy Cabin* (Easy Cabin, 2021) is a modular solution for leisure living and remote working	The solution adapts to different needs, from a guest cottage to a remote workspace or a sauna to a terrace extension	The cost of the solutions ranges from 6 to 45 k€	The solution assembly is quick, and foundations and site work are not needed, which is environmentally friendly as C0_2_ is not released into the air. The solution uses wood as a building material. The solution is reusable, energy-efficient, and has a long lifecycle
*Apartments that allow either multifunctionality or uniting or separating two apartments*			
*Duo apartments* (Bonava Oy, 2021) are larger family apartments (82–98 m^2^) that consist of two complementary apartments that can be united or separated	The apartment empowers the resident to set aside a separate studio for, e.g., a young person that wants to become independent, an older person, or for rental use	The apartment is a bit more expensive than a similar size apartment without the flexibility option. Both apartments have their own share groups, so it is possible to sell them separately	The apartment buildings meet the current Finnish building act
*Heartwood* (Lakea Oy, 2017) concept unites concrete and wood construction for apartment buildings. The concrete bathroom element is located in the middle of the building, and the wooden elements are assembled around it. The wooden elements can be flexibly placed, allowing some adaptability. The apartments can be connected or separated by increasing or decreasing the openings between the elements	The concept empowers the resident to increase or decrease the size of the apartment	No mention of the cost Target group: apartments can be rented, owned, or the resident can gradually become the owner of the apartment by paying rent through the concept “Omaksi” (to your possession)	The wooden elements absorb carbon dioxide and are industrially manufactured, which reduces construction waste and material usage. As a result, the carbon footprint is at least half that of a stone apartment building
*SATO’s flexible small apartments* (Hammarsten, 2019) new studio homes are no longer bounded by load-bearing walls at all, thus allowing for flexibility	Meets changing space needs	Rental apartments Target group: adults	In a couple of years, all new studios will be flexible and can be combined with neighboring apartments. There will be lightweight partitions that can be removed or a door made into them
*Domestic Transformer* (EDGE Design Institute Ltd, 2016) in Hong Kong is a 32 m^2^ space that transforms to support various functions (bathroom, kitchen, living room) inside an apartment building. The home is controlled with a smartphone	Meets various living functions	There is no mention of the cost; probably quite expensive, as the solutions necessitate structural changes Target group: wealthy adults	A resource-efficient concept as the space supports various functionalities in the home

Most of the identified small houses are built from wood, supporting environmental sustainability. Some house models have even solar as an energy source. The house models range from small student housing to cottages and detached houses. Most of them empower the resident to extend their space. As a result, the prices of the modules also range from 6 k€ to several hundred k€. Some concepts allow renting of the house.

The identified apartments can be either rented or bought. Some apartments include moving walls or sliding partitions to divide spaces. Currently, smaller apartments are built, but they include solutions supporting flexibility, such as movable storage solutions and fold-out beds. In addition, some apartments allow either multifunctionality or uniting or separating two apartments. The sustainability of these apartments is hard to judge; some of them use wood as a construction material. All of them, of course, meet the current Finnish building act.

Empirical studies have not examined demand preferences for portable dwellings (Glumac, 2021). Still, mini/tiny homes might gain popularity in the future as land prices are growing, but many people do not want to live far away from the city centers and have big bank loans. For example, some small farm villages in Finland have started to design their own mini homes (Maaseudun sivistysliitto, 2021). Mini homes fit into tiny spots; city zoning is one of the factors that can determine whether these kinds of homes can be built near cities. Still, it is rare, also globally, to encounter proposals to assemble tiny homes into viable communities so that it would be possible to realize reductions in direct energy use and improved sustainability performance due to population density (Cohen, 2021).

As concluded by (PwC & the Urban Land Institute, 2022), repurposing typical (normal) buildings is still challenging to execute. Flexible house solutions can make this easier. The US mobile homes do not require a foundation or a basement, and thus they can be sited nearly anywhere permitted by the building codes (Aman & Yarnal, 2010). It seems that the identified transferable houses typically share this function. However, building permits vary in Finnish municipalities. Therefore, it is always necessary to review the municipality's practices in question.

Some of the identified flexible and sustainable market solutions, especially tiny houses, support ‘urban compact living’, which is closely connected to social awareness and anti-materialism (Winther, 2021) and sustainability, cost, freedom, and mobility (Mutter, 2013). According to Adabre et al. (2020), in developed countries, the top five critical barriers to sustainability attainment in affordable housing include inadequate affordable housing policy, inadequate public funding, income inequality, income segregation, and high cost of service land. The identified house solutions do not directly respond to any of these barriers.

According to Häkkinen and Belloni (2011), sustainable buildings are not prevented by a lack of technologies or assessment methods but due to a lack of adequate organizational procedures for adopting new methods. However, the conclusion could be different today, given the greater importance of sustainability than during Häkkinen’s and Belloni’s study (Häkkinen & Belloni, 2011). This is supported by the fact that all market solutions can at least somehow be assessed from a sustainability perspective, and they can be seen promoting UN’s wider Sustainable Development Goals 11 and 12 (United Nations, n.d.). However, there may be tradeoffs among different sustainability objectives (Karatas & El-Rayes, 2015), leading to challenges in design.

## Conclusions

This study dealt with motivations and market solutions for housing flexibility in Finland. The research questions addressed: (1) trends driving housing flexibility, (2) views on flexible housing, and (3) existing market solutions for flexible and sustainable housing. In the following, we summarize the findings and, after that, provide conclusions.

First, we found that housing-related trends driving towards flexibility exist, although no evidence of flexibility as a separate housing trend was found. For example, extending the service lifetime of buildings, remote working, downsizing of physical belongings, and concerns about housing affordability are trends having connections to flexible housing. Furthermore, there exist market solutions for each trend that somehow support housing flexibility (see Table 3).

**Fig. 7:**
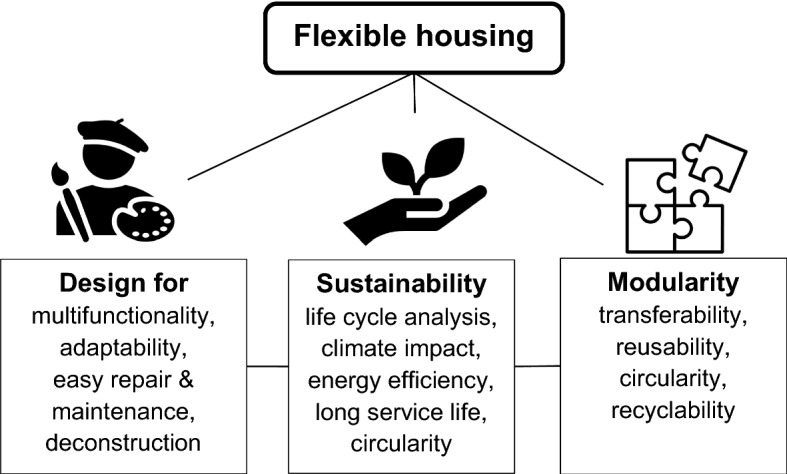
Topics related to flexible housing

Second, according to the interviewees’ views, flexibility in housing means being able to use the premises according to current needs. Although there is a certain consensus on what flexibility in housing means, the diversity of the concept emerged in the interviews. Flexibility is an umbrella term for various concepts—such as adaptability, convertibility, versatility, multifunctionality, modularity, and transferability—which are means to provide flexible housing in practice. Furthermore, sustainability is also related to flexibility through the long service life of buildings and the circularity of building materials. Flexibility also provides the opportunity for increasing or decreasing housing space when needed. Depending on the situation, flexibility may even unite different generations if two apartments can be united or separated. Finally, housing flexibility also allows the owner to move certain decisions to a later stage. Figure 7 summarizes the different views in the interviews and the results from the trend search.

No insurmountable technical challenges for housing flexibility were found, even if building services flexibility is complex. Flexibility between two dwellings necessitates double reservations for building services. In addition, housing flexibility increases the design and construction costs somewhat. According to the stakeholder interviews, integrating two apartments is challenging and costs more than buying a bigger one.

Flexible housing solutions provide companies with the possibility to widen their product offering. When designing housing flexibility, certain issues need to be locked early in the planning process. However, housing flexibility also allows the owner to postpone certain decisions. For example, it may allow transforming a closet into a sauna later if the layout is designed to support it.

According to the interviews, the market demand for flexible housing solutions in Finland is low. In part, this is because housing is often thought of only in terms of the current life situation. However, market demand may increase if awareness of flexible options increases. Furthermore, the interviewees mentioned several market solutions that provide flexibility. For example, there are ways to provide flexibility within an apartment as well as implementation solutions for connecting two apartments.

Third, the reviewed market solutions could be classified under two categories, namely, (1) Small, transferable houses built in a factory and assembled at the site or the factory and (2) Apartments allowing either multifunctionality or uniting or separating two apartments (See Table 4). Most of these houses in the first category are built from wood, which supports environmental sustainability. The models range from small student housing to cottages and detached houses. Most of them empower the resident to extend their space. As a result, the prices of the modules also range from 6 k€ to several hundred k€. Some concepts allow renting the house. The apartments in the second category can be either rented or bought. Some apartments include moving walls or sliding partitions to divide spaces. Currently, it seems that smaller apartments are built, but they include solutions supporting flexibility. In addition, more and more eco-friendly modular construction is used to build these apartment buildings. Overall, the sustainability-related information in both categories was limited. Finally, our results provide the city policymakers with empirical evidence that flexible housing solutions exist, but various aspects still need to be developed, including marketing, readiness, recyclability, easiness, and sustainability.

As in all studies, this one also includes limitations. One of them is the low number of interviewees. To alleviate this limitation, we used different data sources for data triangulation. We also conducted the interviews and their analysis together to increase validity. In addition, the study does not aim for a wider generalization but looks at housing flexibility with a broad scope. Another limitation of the study is that it mainly focuses on market solutions for housing flexibility in Finland. However, we have also added some examples abroad to alleviate this limitation. The third limitation is that we did not interview dwellers. However, we searched for studies of Finnish housing preferences. These studies are referred to in Sect. 4.2.4. Finally, we did interview all other stakeholders in the value chain, including construction companies, designers, housing providers, financers and regulatory authorities.

Based on our findings, we propose that future research investigates how residents consider flexible housing and what kinds of solutions they consider desirable, acceptable and sustainable. Also, it would be beneficial to understand how virtual means could increase the flexibility of housing solutions in a user-friendly way. Furthermore, it is crucial that future R&D on flexible housing is conducted in cooperation with the construction industry and other market actors. Finally, better insights are needed to understand how building regulations and building permit processes support and restrict relocatable buildings and how they should be developed.

## Data Availability

The datasets generated during and/or analysed during the current study are not publicly available due to GDPR restrictions and privacy promised to the interviewees in the informed consent, but parts of data are available from the corresponding author on reasonable request.

## Appendix

### Background information about the interviewee

- Name
- Organization
- Role in the organization
- Years in the organization
- Years of experience

### Flexibility during housing use and its benefits

- What does flexibility during housing use mean to your organization?
- How does flexibility during housing use thought in your organization?
- What benefits (/have you got/could you get) from the flexibility in housing?

### Challenges related to increasing housing flexibility

- What economic factors do you think hinder the flexibility in housing? Why?
- What do you think are the biggest technical challenges for the flexibility of housing during use? Why?
- What are the other challenges to housing flexibility?

### Market readiness for flexible modular housing solutions

- What kind of flexible modular housing solutions have you encountered in the market?
- How do you rate them? (economy, functionality, environmental aspects)
- How could this market be promoted?
- Is there anything else in your mind that you would like to tell us?

## Code availability

Research material other than that contained in the manuscript is not openly available.
